# Influence of Thermal Annealing on the Sinterability of Different Grades of Polylactide Microspheres Dedicated for Laser Sintering

**DOI:** 10.3390/ma14112999

**Published:** 2021-06-01

**Authors:** Małgorzata Gazińska, Anna Krokos, Bartłomiej Kryszak, Paulina Dzienny, Michał Olejarczyk, Piotr Gruber, Ryszard Kwiatkowski, Arkadiusz Antończak

**Affiliations:** 1 Faculty of Chemistry, Department of Engineering and Technology of Polymers, Wroclaw University of Science and Technology, Wyb. Wyspiańskiego 27, 50-370 Wrocław, Poland; anna.krokos@pwr.edu.pl (A.K.); bartlomiej.kryszak@pwr.edu.pl (B.K.); 2 Laser & Fibre Electronics Group, Faculty of Electronics, Wroclaw University of Science and Technology, Wyb. Wyspiańskiego 27, 50-370 Wrocław, Poland; paulina.dzienny@pwr.edu.pl (P.D.); arkadiusz.antonczak@pwr.edu.pl (A.A.); 3 Centre of Advanced Manufacturing Technologies, Faculty of Mechanical Engineering, Wroclaw University of Science and Technology, Wyb. Wyspiańskiego 27, 50-370 Wrocław, Poland; michal.olejarczyk@pwr.edu.pl (M.O.); piotr.gruber@pwr.edu.pl (P.G.); 4 Institute of Textile Engineering and Polymer Materials, University of Bielsko-Biała, Willowa 2, 43-309 Bielsko-Biała, Poland; rkwiatkowski@ath.bielsko.pl

**Keywords:** polylactide microspheres, thermal conditioning, sintering window, laser sintering, powder morphology and flowability, crystalline structure, additive manufacturing

## Abstract

We present a comparison of the influence of the conditioning temperature of microspheres made of medical grade poly(L-lactide) (PLLA) and polylactide with 4 wt % of D-lactide content (PLA) on the thermal and structural properties. The microspheres were fabricated using the solid-in-oil-in-water method for applications in additive manufacturing. The microspheres were annealed below the glass transition temperature (T_g_), above T_g_ but below the onset of cold crystallization, and at two temperatures selected from the range of cold crystallization corresponding to the crystallization of the α’ and α form of poly(L-lactide), i.e., at 40, 70, 90, and 120 °C, in order to verify the influence of the conditioning temperature on the sinterability of the microspheres set as the sintering window (SW). Based on differential scanning calorimetry measurements, the SWs of the microspheres were evaluated with consideration of the existence of cold crystallization and reorganization of crystal polymorphs. The results indicated that the conditioning temperature influenced the availability and range of the SWs depending on the D-lactide presence. We postulate the need for an individual approach for polylactide powders in determining the SW as a temperature range free of any thermal events. We also characterized other core powder characteristics, such as the residual solvent content, morphology, particle size distribution, powder flowability, and thermal conductivity, as key properties for successful laser sintering. The microspheres were close to spheres, and the size of the microspheres was below 100 µm. The residual solvent content decreased with the increase of the annealing temperature. The thermal conductivities were 0.073 and 0.064 W/mK for PLA and PLLA microspheres, respectively, and this depended on the spherical shape of the microspheres. The wide angle X-ray diffraction (WAXD) studies proved that an increase in the conditioning temperature caused a slight increase in the crystallinity degree for PLLA microspheres and a clear increase in crystallization for the PLA microspheres.

## 1. Introduction

Laser sintering is a powder bed fusion additive manufacturing technology in which preheated polymer material in the form of a powder is fused together using laser radiation. A layer-by-layer process of melting and solidification of the radiation-subjected cross-section results in the manufacture of a three-dimensional part. The most commonly used materials in laser sintering (LS) technology are semicrystalline polymers; however, amorphous polymers or even glass and ceramics can be used. Applicable polymer powders in LS must fulfil several conditions, such as appropriate particle shape, size distribution, and thermal, rheological, and optical properties [1]. Only particularly controlled property combinations will lead to successful LS implementation.

In terms of the thermal properties of semicrystalline polymers, crystallization during processing should be avoided. Thus, the processing temperature must be set between melting and crystallization—the temperature range called the super cooling window, which corresponds to the sintering window (SW) [1]. This standard definition of the SW for LS processing works for polyamide (PA11 and PA12), which exhibits a sharp melting peak and broad supercooling range [2]. In the case of amorphous polymers, the processing temperature is evaluated differently [3]. The most common powder processing temperature is set close to the glass transition temperature (T_g_), which allows for stress relaxation and reduces the warpage of produced parts [4,5,6] and is still high, which results in brittle and unstable parts due to improper powder particle coalescence [7,8].

Another approach concerning SW determination was proposed by Berretta et al. [9] for high-temperature semicrystalline polymers, which do not show a super-cooling window, such as poly(ether ether ketone) (PEEK) and poly(aryl ether ketone) (PEAK). The method involves a calculation of the first derivative of the heating curve of a differential scanning calorimetry (DSC) thermogram and then calculating the minimum point and was proven to correspond with the actual powder processing temperatures for tested high-temperature polymers. However, this technique still needs to be fully validated with a wider range of materials.

Additive manufacturing is increasingly used in the healthcare sector in applications, such as anatomical models, medical devices, pharmaceuticals, and tissue engineering [10]. Biomaterials processed with LS that are used or being implemented (on a different stage of development) in the medical industry are PEEK [11,12], polycaprolactone (PCL) [11,13,14], poly(vinyl alcohol) (PVA) [11,15,16], polylactide with 4 wt % of D-lactide content (PLA) [17], and poly(L-lactide) (PLLA) [11,13]. The SW should ensure that the powder lying in the powder bed of a generic LS system does not melt before exposure to the laser and does not crystallize before or during laser exposure.

The SW defined as a super cooling window for semicrystalline polymers is a proper for example for polyamides. However, the group of semicrystalline polymers processed by LS includes polymers that crystallize relatively slowly and undergo so-called cold crystallization during heating. These polymers are poly(ethyl terephthalate) (PET), PLLA, polycarbonate (PC), and PEEK. Often, in the case of PLLA, cold crystallization is neglected and the printing parameters—i.e., the bed temperature—are selected regardless of whether or not cold crystallization will occur [13,18].

If the PLLA powder has a low degree of crystallinity, cold crystallization may occur in the powder bed. As a consequence of having a temperature gradient in the powder bed [19,20,21] the polylactide powder could have spatial crystallinity degree distribution. Therefore, it is important to determine the SW taking into account cold crystallization. An alternative solution is additional conditioning before sintering, in order to obtain completely crystallized powder.

M. Van den Eyden at al. described the influence of thermal treatment on the laser sinterability of polybutene-1 (PB-1). The authors demonstrated two alternative thermal treatment strategies leading to broadening of the SW of PB-1 and making an unsinterable polymer sinterable [22].

In the scope of this article, we focus on the determination of the thermal and structural properties of microspheres made of two types of polylactides. Medical grade poly(L-lactide) (PLLA) and a polylactide (PLA) stereocopolymer with 4% D-isomer content were intentionally selected due to their extremely different crystallization behaviors [23].

The principal aim was to elucidate the influence of the conditioning temperature on the thermal and structural properties of the microspheres in order to control the availability and range of SW. The degree of crystallinity and α/α’ crystal form of poly(L-lactide) was determined for the PLLA and PLA microspheres annealed at different temperatures. The crystal form of PLLA significantly affects the application properties, such as the mechanical and barrier properties [24,25]. In addition, as for degradable polymers, the degradability of PLLA is also influenced by polymorphism. N. Zhang et al. demonstrated different hydrolytic degradation behaviors of α’- and α-PLLA [26]. Thus, control of the polymorphism is important for optimizing the properties of PLLA for biomedical applications.

In addition, the goal of the research was the precise determination and definition of a processing window of different grades of polylactide microspheres as an example of a semicrystalline polymer that exhibits cold crystallization. The SW was determined based on DSC curves recorded at a standard rate of 10 °C/min. Due to the crystal polymorphism of PLLA, the reorganization of conformationally disordered α′-crystals into stable α-crystals occurs on heating. In our previous paper, we precisely describe the SW determination for PLLA and composite microspheres consisted of PLLA and hydroxyapatite particles [27]. That research was focused on the influence of HAP particles on the SW range.

In the processing window, the sinterability of the polylactide microspheres was verified based on the morphology, particle size distribution, powder flowability, thermal stability, and residual solvent content.

## 2. Materials and Methods

### 2.1. Materials

The two types of polylactides used in this study were commercial products, including polylactide PLA 3051D grade (4% D-lactide content, inherent viscosity 3.0–3.5 dL/g) from Nature Works (Nature Works LLC, Minnetonka, MN, USA) and medical grade poly(L-lactide) PLLA Resomer L207S (inherent viscosity 1.5–2.0 dL/g) from Evonik (Evonik Industries AG, Essen, Germany). Methylene chloride (CH_2_Cl_2_) was supplied from STANLAB (Lublin, Poland), and poly(vinyl alcohol) (Mowiol 18–88, Mw~130,000, 86.7–88.7 mol % hydrolysis) from Sigma-Aldrich (Darmstadt, Germany)was used in our research.

### 2.2. Preparation of PLA and PLLA Microspheres

Microspheres of PLA and PLLA were prepared using the conventional emulsion-solvent evaporation technique (O/W). At first, a 1% aqueous solution of PVA was prepared by stirring with a magnetic stirrer (Heidolph Instruments GmbH & Co. KG, Schwabach, Germany) and heating up to 70 °C. Then both PLA and PLLA were dissolved in methylene chloride (CH_2_Cl_2_) to obtain the 4 wt % transparent solution. The volume ratio of organic phase to aqueous phase was 1:3. Next, the organic solution was dropped into PVA solution by using a peristaltic pump (Zalimp, Warsaw, Poland) while the PVA solution was stirred with magnetic stirring at 800 rpm at room temperature. After emulsification, the methylene chloride was removed by evaporation at room temperature under stirring over 48 h. PLA and PLLA microspheres were separated by centrifuging and drying in a vacuum at 40 °C for 24 h. After preparation, the microspheres were additionally annealed at 40, 70, 90, and 120 °C for 5 h. The series of microspheres were abbreviated PLA_T and PLLA_T, where T stands for the conditioning temperature.

### 2.3. Scanning Electron Microscopy

Microspheres of PLA and PLLA were characterized using scanning electron microscopy (SEM) using Zeiss EVO MA25 (Zeiss, Oberkochen, Germany) with the back -scattered electron detector and accelerating voltage of 20 kV. The captured SEM using Zeiss EVO MA25 images allowed us to determine the shape and size of the investigated powders.

### 2.4. Particle Size Distribution

The particle size distribution of the prepared powders was determined by dry laser diffraction spectroscopy using HELOS/BR 4470 C, RODOS/T4, R4 with a measurement range of 0.1–875 µm according to the ISO 13220-1 standard. The dispersing pressure was set at 2 bar along with a VIBRI feeder (Sympatec, Clausthal-Zellerfeld, Germany) with a feed rate of 80% and gap width of 3.5 mm. The particle size, D_50_, was determined to represent a powder particle diameter where 50% by volume of the powder particles were smaller. Additionally, the d_10_, d_90,_ and percentage of particles below 10 µm were established. The d_10_ and d_90_ were used to calculate the span, as (1)

Span = (d_90_ − d_10_)/d_50_ (1)

### 2.5. Powder Flowability

The dynamic powder flowability was determined using the Revolution Powder Analysis (RPA) method. A drum (GranuDrum, GranuTools, Awans, Belgium) with an inner diameter of 84 mm and a 20 mm width rotates around its axis at an angular velocity ranging from 2 to 60 rpm. The transparent sidewalls allow for observation of the powder behavior inside, which can be captured by the image vision system [28]. This method allows for the measurement of the first avalanche angle (AA), flowing angle (α_f_), and dynamic cohesive index (σ_f_). The avalanche angle describes the angle at which the powder reaches the highest potential energy (the highest point) in the drum just before the loss of stability, which is visible as an avalanche [8].

The avalanche behavior of a powder sample can be used as a good predictor of the powder flowability for powders that are not too cohesive [29]. The first avalanche angle is measured by image analysis at a low rotational speed, and the flowing angle is measured at a variable rotational speed. In general, a low value of these factors corresponds to a good flowability [30,31]. In this test, one rotating speed (1 rpm) for the first avalanche angle and nine different rotating speeds between 2 and 60 rpm were used, both at increasing and decreasing angular velocity for the flowing angle. We took 25 images for each rotation speed, separated by 1 s.

Based on the recorded images, the average position of the powder/air interface and the fluctuations around this value were tracked. The dynamic cohesive index is related only to the cohesive forces acting between the grains [30]. We assumed that an increase in the cohesiveness of the powder leads to a corresponding increase in the cohesive index. The drum for each batch was half-filled with powder (roughly 55 mL).

### 2.6. Thermogravimetry

A TGA/DSC1 Mettler Toledo (Greifensee, Switzerland) system was used for the thermogravimetric analysis (TGA) of polylactide and poly(L-lactide) microspheres for estimation of the residual solvent content and thermal stability of the microspheres. The samples were heated at the rate of 10 °C min^−1^ from 25 to 650 °C under 60 mL/min of nitrogen flow.

### 2.7. Differential Scanning Calorimetry

Differential scanning calorimetry (DSC) measurements were performed using the Mettler Toledo DSC1 (Greifensee, Switzerland) system, coupled with a Huber TC 100 intracooler (Offenburg, Germany). The instrument was calibrated using indium (T_m_ = 156.6 °C, ΔH_m_ = 28.45 J/g) and zinc (T_m_ = 419.7 °C, ΔH_m_ = 107.0 J/g) standards. Samples (~3.5 mg) were measured in 40 µL aluminum pans under a constant nitrogen purge (60 mL/min) from 0 to 200 °C. The heating rate was set to 10 °C/min. DSC curves for the estimation of the thermal conductivity of microspheres were recorded from 150 to 170 °C with a heating rate of 0.5 K/min under 150 mL/min nitrogen flow. The DSC curves were normalized to the sample mass. The evaluation of the thermal properties from the DSC and TGA curves was performed using the STARe software (16.20c version). The initial degree of crystallinity (X_c_) of PLLA was calculated from the first heating DSC curves according to Equation (2)

(2) $$X_{c}^{\mathrm{DSC}}=\frac{∆H_{m}-∆H_{\mathrm{cc}}}{∆H_{m}^{100\%}},$$

where ΔH_m_ is the melting enthalpy [J/g], ΔH_cc_ is the enthalpy of cold crystallization, ΔH_m_^100%^ is the melting enthalpy of the α’-form of 100% crystalline polylactide (107 J/g) and the melting enthalpy of the α-form of 100% crystalline polylactide (143 J/g) [32].

### 2.8. Wide Angle X-ray Diffraction

Wide angle X-ray diffraction (WAXD) experiments were performed at room temperature on a Rigaku Ultima IV diffractometer (Bragg–Brentano geometry) (Rigaku International Corporation, Tokyo, Japan) with Ni filtered Cu Kα (λ = 1.54178 Å) radiation generated by a sealed X-ray tube. The radiation source was powered by a generator operated at 40 kV and 30 mA. The data were collected within the range of 2θ from 1.5° to 65.0° in a continuous scan mode with a step width of 0.005° and a scanning rate of 5 °C/min. The background corrected WAXD patterns were resolved into Lorenzian shape diffraction peaks and diffusion maxima by using the Levenber–Marquardt non-linear fitting procedure implemented on OriginPro 9.0 software. The degree of crystallinity was calculated according to the following Equation (3)

(3) $$X_{c}^{\mathrm{WAXD}}=\frac{\sum^{\;}A_{c}}{\sum^{\;}A_{c}+\sum^{\;}A_{a}}\cdot 100\%,$$

where A_c_ and A_a_ represent the integrated intensities under the crystalline reflections and the integrated intensities under diffuse maxima.

## 3. Results

The analysis of the morphology, particle size distribution, powder flowability, and thermal conductivity was performed for PLA and PLLA microspheres conditioned at 40 °C; whereas, to optimize the crystallinity degree, processing window, and residual solvent content, the XRD, DSC, and TGA analyses were extended to microspheres annealed at higher temperatures (70, 90, and 120 °C).

### 3.1. Scanning Electron Microscopy

**Figure 1:**
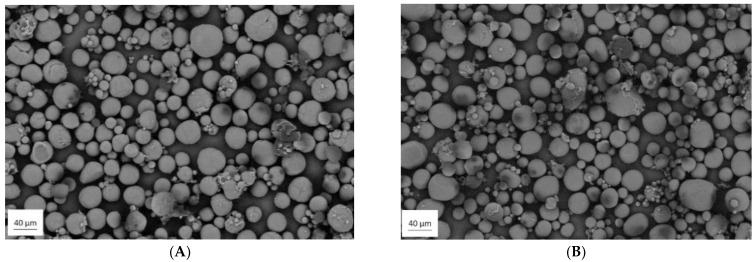
Microscopic images of the powder particles of polylactide with 4 wt % of D-lactide content (PLA_40) (**A**) and poly(L-lactide) (PLLA_40) (**B**).

The morphology of the prepared powders is presented in Figure 1. Both PLA_40 and PLLA_40 have a spherical shape with the more homogeneous surface, which is considered as a favorable powder particle shape due to its high flowability and high powder packing density [8]. In addition to the spherical particles in each powder, a small amount of residues in the form of flakes from the preparation process can be observed. Moreover, the morphology of the PLA and PLLA powders annealed at 70, 90, and 120 °C does not change as steam from SEM images combined in Figure S1 (in Supplementary Materials). SEM images confirmed that the powders were suitable for the LS process.

### 3.2. Particle Size Distribution

**Figure 2:**
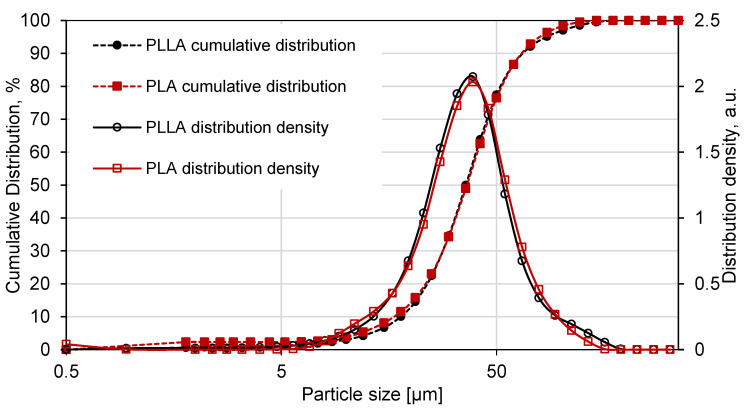
Cumulative distribution and distribution density of PLA and PLLA.

As measured by dry laser diffraction spectroscopy, the powder’s properties are shown in Table 1. The reported particle size distribution (PSD) used in commercial LS systems should be up to 200 μm, which, in practice, is commonly in the range of 20 to 80 μm [33]. The lower limit of particle size is commonly presented as 10 μm due to the negative influence on the bulk flow at high temperatures [34]. The cumulative particle size distribution is shown in Figure 2, and both material distributions can be characterized as narrow, symmetrical, and unimodal.

### 3.3. Powder Flowability

**Figure 3:**
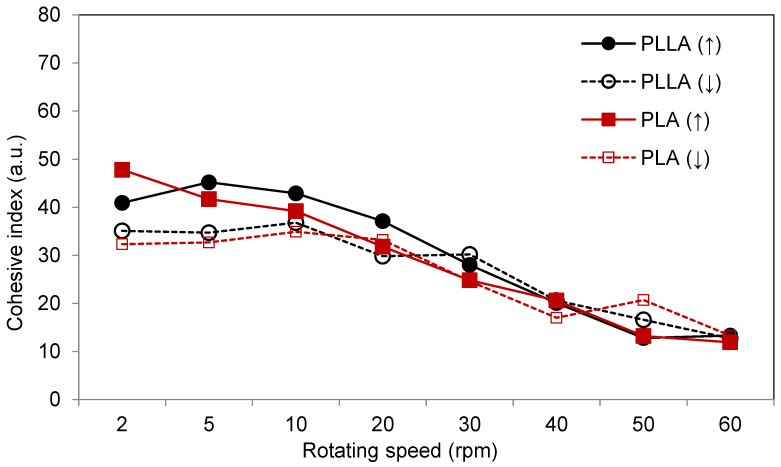
Cohesive index curves recorded as a function of the increasing (↑) and decreasing (↓) rotation speed for PLA and PLLA powders.

The basic flowability parameters are shown in Table 1, and the dynamic flowability represented by cohesion index curves as a function of rotating speed are presented in Figure 3. The avalanche angle, which is the first angle that triggers the pouring of powder, provides information indicating that, in the quasi-static conditions, PLLA had a slightly better flowability. These differences may be caused by the lower percentage of particles below 10 μm. However, when looking at dynamic flowability and the cohesive index, especially for lower velocities (up to 20 rpm), one can see that the flowability properties of PLA were slightly better. Better dynamic flowability properties are represented by smaller cohesive index. The small differences in powders’ flowability are expected, especially considering similar powder morphonology and particle size distribution.

### 3.4. Residual Solvent Content and Thermal Stability of Microspheres

Based on the TGA curves of the PLA and PLLA microspheres presented in Figure S2 (in the Supplementary Materials), the residual solvent content as a mass loss in the range up to 200 °C and the thermal stability corresponding to 5 wt % of the mass loss (T_−5%_) were determined. The data collected in Table 2 demonstrates that with the increasing annealing temperature of microspheres, the content of residual solvents (water and methylene chloride) in microspheres of PLA and PLLA decreased. Annealing at higher temperatures led to faster rates of solvent removal from the microspheres. The residual solvent content decreased from 0.49 to 0.02 wt % for PLA and from 0.25 to 0.02 wt % for PLLA microspheres with the increase of the annealing temperature.

The residual solvent content in the microspheres of PLA was significantly lower after the drying process at 70 °C compared with at 40 °C, whereas the microspheres of PLLA required a higher drying temperature. The residual solvent and adsorbed/absorbed water content must be removed to minimize the agglomeration of microspheres and the emission of toxic substances during the laser sintering process [8]. The CH_2_Cl_2_ belongs to class 1 of residual solvents in pharmaceuticals according to the classification of residual solvents by risk assessment.

If their use in order to produce a medicinal product is unavoidable, their levels should be restricted because of the unacceptable toxicities [35]. Due to these two aspects, such as the safety of LS processing and biomedical applications of “printed” details, the content of residual CH_2_Cl_2_ should be qualified as a standard control parameter and must be kept to the lowest possible level. The thermal stability of both types of microspheres rose slightly with the increasing conditioning temperature reaching T_−5%_ higher at about 3 °C for PLA and 4 °C for PLLA microspheres annealed at 120 °C.

### 3.5. Thermal Properties of Microspheres

DSC analysis was performed to characterize the thermal properties of the microspheres as prepared and to determine the influence of the conditioning temperature. The main purposes were to determine the temperature range of the processing window and verification of the possibility of tuning the SW through conditioning of the microspheres.

**Figure 4:**
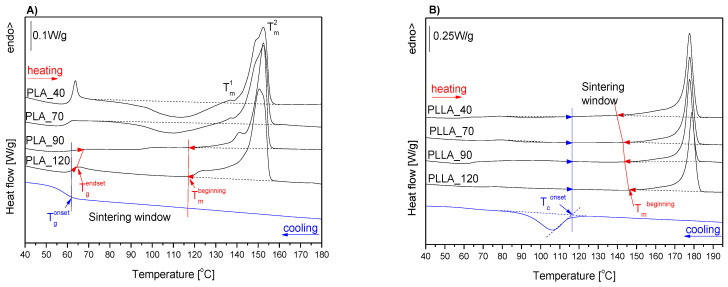
First heating and cooling DSC curves of PLA (**A**) and PLLA (**B**) microspheres annealed at 40, 70, 90, and 120 °C. For simplicity, only one cooling curve recorded after the complete melting and isotropization of PLA and PLLA microspheres is presented on the A and B graphs, respectively. The cooling curves of the series of PLA as well as PLLA microspheres were similar within series independently regarding the annealing temperature (Figure S3 in the Supplementary Materials).

The first heating and cooling DSC curves of PLA microspheres are presented in Figure 4A, and the evaluated thermal properties are collected in Table 3. Based on comparison of the first heating DSC curves of PLA microspheres, the curing temperature influenced the thermal properties of PLA, with the strongest differences concerning the cold crystallization behavior and crystallinity degree. Microspheres annealed at 40 and 70 °C during heating crystallized above the glass transition temperature. The enthalpy of cold crystallization (ΔH_cc_) of PLA_40 was higher than PLA_70, indicating a higher content of the crystalline phase of PLA_70 microspheres compared with PLA_40.

Microspheres PLA annealed at 90 and 120 °C reached the maximum degree of crystallinity during conditioning, and this was confirmed by the lack of exotherm of cold crystallization during heating with the rate of 10 °C/min. At higher temperatures, endothermic melting was visible on the DSC curves of the PLA microspheres with the peak temperature at 152 °C. The microspheres of PLA_120 had different trace melting peaks, compared to other PLA microspheres. The melting endotherms of PLA_40, PLA_70, and PLA_90 exhibited two maxima, denoted as T_m_^1^ for the lower temperature maximum and T_m_^2^ for the main melting maximum. The presence of a small endothermic maximum on the leading edge of the main melting peak indicates the presence of a mixture of α’ and α crystals. There is a clearly visible trend of T_m_^1^ decreasing with decreasing of the conditioning temperature due to the lower stability of crystals that were formed at higher supercooling temperatures [36].

One of the key purposes of DSC measurements was determination of the temperature range of the processing window. Typically, the SW for semicrystalline polymers is defined as the temperature band between the onset of melting (T_m_^onset^) and the onset of melt crystallization (T_c_^onset^). The presence of cold crystallization had a significant impact on the temperature range of the processing window. In the case of PLA_40, a broad range of cold crystallization—starting above the enthalpy relaxation effect, overlapping on the glass transition, and continuing up to melting—indicates the lack of a processing window and should exclude these microspheres from any thermal processing.

Similarly, the microspheres of PLA_70, although the cold crystallization started at higher temperature, were also not suitable for laser sintering. Based on the DSC curves of PLA_90 and PLA_120, we concluded that, due to the crystallization that occurred during conditioning at 90 and 120 °C, the microspheres did not undergo cold crystallization upon subsequent heating. Thus, due to the conditioning at 90 and 120 °C, the PLA microspheres demonstrated a processing window. The PLA microspheres under cooling did not crystallize from melt, as shown in the cooling DSC curve (Figure 4A); thus, the lower limit of the SW can be defined as the onset of the glass transition (T_g_^onset^).

The T_g_^onset^ designated from the cooling DSC curve equaled 63 °C for PLA microspheres. However, as depicted in Figure 4A, the T_g_^onset^ estimated from the cooling DSC curve was lower than endset of the glass transition (T_g_^endset^) from the heating scan (65 and 67 °C for PLA_90 and PLA_120, respectively). The T_g_^endset^ is defined as the extrapolated endset temperature of the glass transition and indicates the end temperature of the glass transition, where the heat capacity dependence becomes linear. Because the processing temperature should start above the glass transition, we emphasize the necessity to use the T_g_^endset^ from the heating DSC curve as the lower limit of SW as more correct than the T_g_^onset^ from cooling. Therefore, the SW was set as the temperature range between T_g_^endset^ and the onset of melting (T_m_^onset^) for PLA_90 and PLA_120 within the ranges of 65–146 °C and 67–144 °C, respectively.

The first heating DSC curves of PLLA microspheres conditioned at different temperatures are presented in Figure 4B. Compared to PLA microspheres, they had an approximately 26 °C higher melting temperature, higher melting enthalpy, and degree of crystallinity, due to differences in the enantiomeric isomer (L-lactide and D-lactide) content. PLLA microspheres crystallized from the melt in contrast to PLA microspheres as was visible on the cooling DSC curve.

In contradiction to the PLA microspheres, the conditioning temperature did not significantly influence the PLLA microsphere thermal properties (Figure 4B). A weak exothermic effect of cold crystallization was visible only on the DSC curves of PLLA_40 and PLLA_70 (∆H_cc_ = 0.7 J/g and 1.8 J/g for PLLA_40 and PLLA_70, respectively). The temperature range of cold crystallization was narrower than for PLA_40 and PLA_70, and, thus, for PLLA microspheres annealed at 40 and 70 °C, the SW was available in contrast to the respective PLA microspheres.

The processing window according to typically used definitions (SW^onset^), set between the onset of melt crystallization (T_c_^onset^) and the onset of melting, was within the range of 116–174 °C for PLLA_40 and differed maximally by about 1 °C due to the T_m_^onset^ in the range of 174–175 °C in PLLA microsphere series.

The high temperature limit of the SW is typically defined as the onset of melting. The onset of melting in differential scanning calorimetry is evaluated as the intersection point of the extrapolated baseline prior to the melting transition and the inflectional tangent [37,38]. The additional transition point is sometimes identified, such as the temperature of the first detectable deviation from the interpolated baseline, which we further denoted as T_m_^beginning^ for melting. We proposed the use of T_m_^beginning^, as the temperature for the beginning of the melting endothermic period, in place of the onset [27].

The melting of semicrystalline polymers is a very broad process, and the onset temperature does not take into account the shape of the leading edge of the polymer melting peak. Thus, we proposed to designate the beginning of the melting endothermic period (T_m_^beginning^) as the high temperature limit of the sintering window (SW^beginning^). The high temperature limit of the SW^beginning^ was set as T_m_^beginning^ to allow for consideration of the existence of a broad leading edge of the melting peak, such as in the case of PLLA and PLA microspheres and to cut off any thermal events taking place above T_m_^beginning^ from the sintering window.

In the case of polylactide, at the broad temperature range of melting, reorganization of α’ to α crystal forms [39] can occur, such as in the case of PLA_90 microspheres. Determination of the processing window as a band between T_m_^beginning^ and T_c_^onset^ ensures elimination of the changes in the degree of crystallinity and in the crystalline form of the presintered polylactide powder.

SW^beginning^ was narrower than SW^onset^ and ended at 140, 143, 144, and 146.0 °C for PLLA _40, PLLA_70, PLLA_90, and PLLA_120, respectively. For PLA_90 and PLA_120, the high temperature limits of SW^beginning^ were at 118 and 117 °C, respectively.

**Figure 5:**
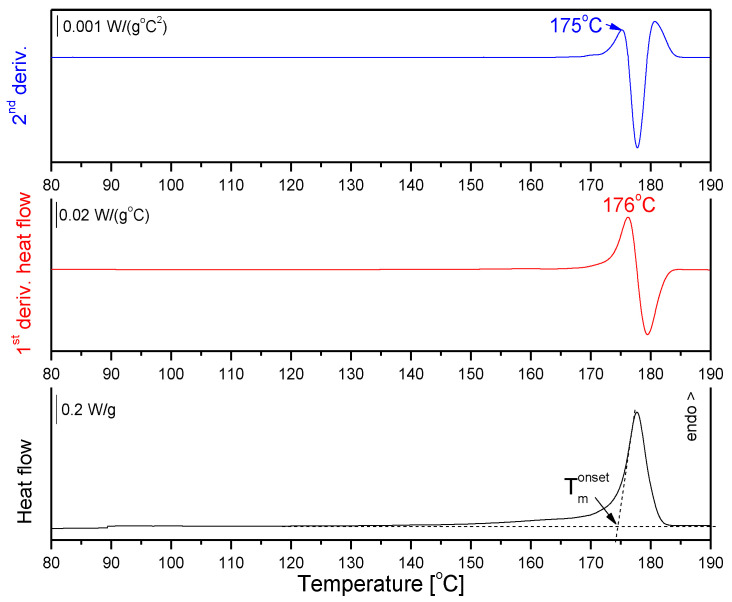
First heating differential scanning calorimetry (DSC) curve of PLLA_90 combined with the first and second derivative of the heat flow.

We also verified the first and second derivatives of the heat flow signal to identify the correct onset of melting transition, as suggested by S. Beretta et al. [9]. The first and second derivatives were automatically evaluated using the ‘Second Derivative’ tool in the STARe software, and are presented in Figure 5 and Figure S4 (in the Supplementary Materials).

The onset of melting taken as the maximum of the first and second derivatives, 176 and 175 °C, respectively, does not exclude the broad leading edge of the endothermic peak of PLLA_90 from the SW (Figure 4). The importance of the correct determination of the upper limit of the sintering window as the beginning of the endothermic period can be seen in the example of the PLA_90 microspheres, as there are two overlapping effects, α’-α reorganization with a maximum at T_m_^1^ and melting with a maximum T_m_^2^ (Figure 4A, Figure S3 in the Supplementary Materials).

The thermal properties of polylactide microspheres, i.e., the SW, were evaluated from DSC curves recorded at 10 °C/min heating and cooling rates, which never exist in LS process. The polymer structure depends on heating and cooling conditioning, and thus, from a practical perspective, the estimated SW should serve as the powder bed temperature. We plan to verify the estimated SW experimentally using a new two-beam laser sintering method. This research will be presented in our next publication.

The influence of the conditioning temperature on the processing window stems from differences in the crystallinity level. Unfortunately, the estimation of the crystallinity degree (X_c_^DSC^) from the DSC results is sometimes not entirely correct. During recordings of the first heating DSC curve, the starting material changed, and cold crystallization took place followed by the reorganization of α’ into the order α phase. The melting enthalpy of 100% crystalline α-crystal PLLA and α’-crystal PLLA were different.

This different values of ΔH_m_^100%^ for the α- and α’-crystals should be considered for the determination of the crystalline content from the experimental melting enthalpies. The estimated X_c_^DSC^ values depend on the ΔH_m_^100%^ (107 J/g and 143 J/g) assumed for the calculation. We made an estimation of X_c_^DSC^ from the DSC results, with more correct X_c_ values, independent of the heat of fusion, determined based on the WAXD results, and they are presented further.

The crystallinity degree (X_c_^DSC^) for the series of PLA microspheres was estimated with taking to account the enthalpy of the melting of the α’-form 100% crystalline polylactide (107 J/g) and the enthalpy of the melting of the α-form 100% crystalline polylactide (143 J/g) for the PLLA microspheres [32]. The presence of the α-form crystals in all PLLA microspheres was indicated by the lack of exothermic effect of the α’-α transition on the first heating DSC curves [39]. For the series of PLA microspheres, based on the broad range of cold crystallization (PLA_40 and PLA_70) and presence of the T_m_^1^ peak (PLA_40, PLA_70, PLA_90) and melting temperature of 152 °C (PLA40, PLA70, PLA90) and 150 °C for PLA120, we assumed that the α’-form is dominant.

As the α’ and α phase precise contents in PLA_40, PLA_70, PLA_90, and PLA_120 are unknown, for simplification of the X_c_^DSC^ calculations, the enthalpy of the 100% crystalline α’-form was employed (Table 3). In the series of PLA microspheres with increasing annealing temperature, the X_c_^DSC^ increased from 23.2% to 41.9%. Whereas, in PLLA microspheres, the crystallinity level was higher but not as strongly dependent on the conditioning temperature.

### 3.6. Thermal Conductivity of Microspheres

Due to the layer-by-layer manner of the LS process, how the polymer absorbs the laser radiation and how the applied heat is transmitted in all spatial directions are both crucial [8]. Schmid et al. reported that a stable layer connection was achievable only if the thermal conductivity of the processed polymer was sufficient to transmit the heat at a depth of at least one previous layer. Jiaming Bai et al. indicated that polyamide 12 with the addition of carbon nanotubes (PA12-CNT) allowed the production of wider and deeper laser penetration compared with powder without additives [40]. Better laser penetration was connected with the greater thermal conductivity of PA12-CNT.

**Figure 6:**
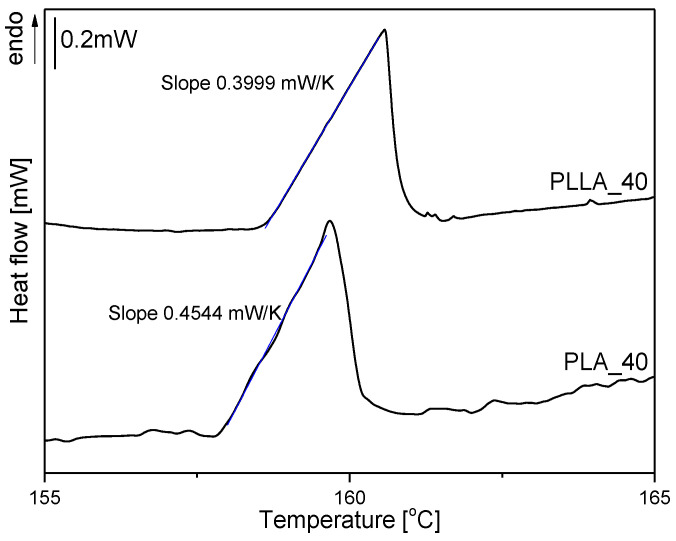
Melting endotherms of indium beads placed on PLA_40 and PLLA_40 microspheres.

The thermal conductivity (κ) of microsphere powders was determined based on DSC measurements according to Camirand’s method [41]. The measurement of κ is made at discrete temperatures that correspond to the melting points of selected pure metal references. As a metal reference, indium was used, and the thermal conductivity of the microspheres was obtained from the slope of the low temperature side of the melting peak of an indium bead placed on top of microspheres filling a 40 µL aluminum pan. The slope of the low temperature side of the indium melting peak depends on the thermal resistance (R) of the measured powder. R is given by the reciprocal of the slope (S). κ was determined from R using Equation (4)

(4) $$\kappa =\frac{\varepsilon }{\pi R}\left(\frac{1}{D_{m}}-\frac{1}{D_{p}}\right)$$

where D_m_ is the diameter of the reference metal bead (1.00 mm), and D_p_ is the diameter of the pan. The value of the constant Ɛ, for a cylindrical aluminum pan with a 5.80 mm diameter and 1.44 mm height, is 1 [42]. For the thermal conductivity of PLA and PLLA microspheres estimation, the DSC curves were measured with nitrogen flow, as the commercially available selective laser sintering (SLS) systems enable processing in an inert atmosphere. The measured DSC curves of indium beads on top of PLA_40 and PLLA_40 microspheres are presented in Figure 6, and the estimated thermal conductivity and resistivity values are collected in Table 4.

The thermal conductivity was 0.073 W/mK for PLA microspheres and 0.064 W/mK for PLLA microspheres. In contrast with their bulk counterparts, the microspheres/powders had very similar conductivities and these were lower than in bulk (0.084 for PLA and 0.104 for PLLA—Figure S5 in the Supplementary Materials). According to S.M. Lebedev et al. [43], the thermal conductivity of neat PLA is 0.193 W/mK. Lu Bai et al. [44] described the impact of the crystallinity of semicrystalline polymers on the thermal conductivity of polymer material and claimed that the thermal conductivity of PLLA increased with increasing crystallinity but not significantly. The thermal conductivity for PLLA with crystallinity of 56% was 0.196 W/mK and 0.171 W/mK for amorphous PLLA.

The thermal conductivity of microspheres differs significantly from solid materials. The lower thermal conductivity of microspheres depends on the spherical shape of the microspheres. Between particles, there are only small contact areas, and air in the pores acts as a thermal isolator. The semicrystalline thermoplastics, such as polyamide 12 (PA12), are commonly used in manufacturing technology related to laser sintering [45]. Alessandro Franco et al. [46] characterized the thermal conductivity of the industrial powder of polyamide 12, which was equal 0.13 W/mK.

Mengqi Yuan et al. [47] also investigated the range of thermal conductivity as 0.09 to 0.12 W/mK for polyamide 12 powder depending of the test temperature, whereas the counterpart in bulk laser sintered polyamide 12 had a thermal conductivity of 0.22 to 0.33 W/mK from 40 to 170 °C, which is approximately three times more than that of the loose powder. The thermal conductivity is a key parameter in the LS of polymer powders. Due to thermal properties of PA12 and our polylactide microspheres, which are appropriate for the thermoplastics used for rapid prototyping, the polyamide 12 powders or polylactide microspheres are suitable materials for the laser sintering processes.

### 3.7. WAXD Analysis

**Figure 7:**
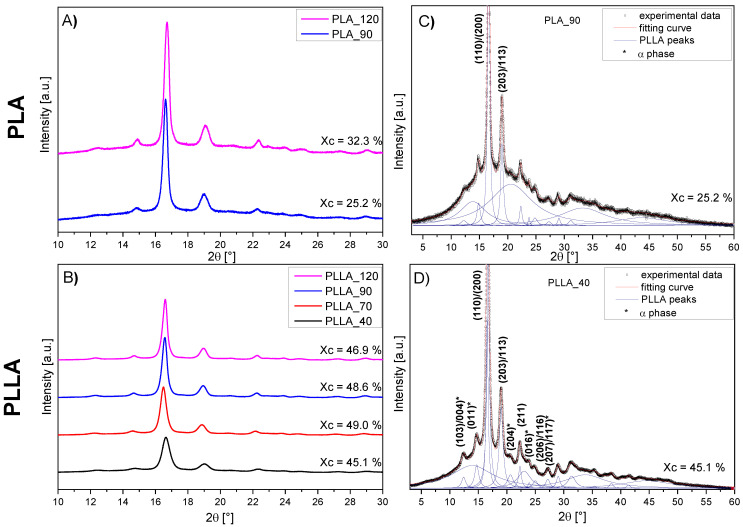
Diffraction curves of the PLA (**A**) and PLLA (**B**) microspheres conditioned at temperatures of 40, 70, 90, and 120 °C. (**C**,**D**) the exemplary deconvolution of curves, respectively of the PLA_90 and PLLA_40.The diffraction peaks marked with asterisks in the graphs A ‘and B’ are characteristic only for the α phase. The others can be connected with both α and α’.

WAXD measurements were performed to assess the crystallinity of PLLA microspheres annealed at different temperatures and PLA microspheres that exhibited SW (PLA_90 and PLA_120). Figure 7 shows a summary of the WAXD diffractograms of the PLA (A) and PLLA (B) microspheres. The course of the curves is characteristic of polylactide [27,32].

A detailed deconvolution of the curves into diffraction peaks and diffusion maxima was made. To perform this procedure, the literature reports were followed [27,48,49]. Figure 7C,D shows two examples for PLLA and PLA powders annealed at 40 °C (a complete breakdown of the deconvolution is provided in the Supplementary Materials in Figure S6). The diffraction maxima on the graph denoted with asterisks (*) corresponds selectively to the crystalline pseudo-orthorhombic, helical α phase of this polymer, while the others are characteristic of both the α and the disordered α’ phase. The expressiveness of these αphase characteristic peaks indicates a significant share of the α phase in the crystal structure of polymers (for both PLLA and PLA) in relation to the disordered α’ phase [32].

The X-ray diffraction study also allowed us to determine the degrees of crystallinity (X_c_^WAXD^) of individual samples. The results of the calculations made are placed above the individual curves and they are consistent with the DSC results. The conducted research showed that an increase in the conditioning temperature caused a slight increase in the polymer crystallinity degree in the case of PLLA. The situation is different for PLA, where the microspheres were initially almost amorphous and clearly crystallized with the increasing annealing temperature.

WAXD studies also confirmed the results obtained in the DSC, indicating the presence of the α’ phase in the PLLA_70 microspheres. This manifested in the shift of the maxima of the main crystalline reflexes toward lower angular positions (by about 0.1°) in relation to the PLA_120 sample.

From comparison of the values of X_c_ calculated from the DSC and WAXD results, certain differences can be noticed. The estimation of crystallinity based on the DSC curve does not correctly reflect the initial crystallinity of the polylactide microspheres, and depends on the assumed heat of fusion as was previously emphasized. The X_c_^WAXD^ values from the WAXD values were in the range of X_c_^DSC^ limit values from the DSC calculated for ΔH_m_^100%^ taken as 107 J/g and 143 J/g. Despite the differences in the X_c_ values from DSC and WAXS, both techniques showed the same trend of the dependence of the crystalline phase content in PLA microspheres on the conditioning temperature, the higher crystallinity of PLLA microspheres, and a slight influence of the conditioning temperature in the PLLA microspheres.

## 4. Conclusions

The aim of the presented research was to determine the influence of the annealing temperature on the thermal properties and crystalline structure of microspheres made of medical grade poly(L-lactide) and polylactide with 4 wt % of the D-lactide content, chosen due to different crystallization behavior. The microspheres were annealed at four temperatures, below the glass transition (at 40 °C), above T_g_ but below the onset of cold crystallization (at 70 °C), and at two temperatures from the range of cold crystallization corresponding to the crystallization of the ɑ’ and ɑ form of poly(L-lactide) (at 90 and 120 °C). The microspheres were dedicated for LS processing; thus, their sinterability was verified based on the SW, residual solvent content, morphology, particle size distribution, and powder flowability.

For the first time the influence of annealing temperatures on the degree of crystallinity, the crystal structure and SW of polylactide microspheres, depending on the polylactide grades, has been presented. In the case of microspheres made of PLA the degree of crystallinity could be precisely controlled due to the lower crystallization ability of PLA compared to PLLA. The presence of a SW of PLA microspheres depended on the crystallinity level. The PLA microspheres annealed at 40 and 70 °C exhibited cold crystallization under heating, and thus they did not have a SW available. However, thermal treatments at higher temperatures made unsinterable PLA microspheres sinterable due to the presence of a SW.

We propose a new definition of sintering window as a temperature range free of any thermal events, which is particularly important for a semicrystalline polymers that exhibit cold crystallization and polymorphism. Any PLLA transformations, such as cold crystallization and reorganization of the crystal structure, should be excluded from the processing window, as the α and α’ crystal forms of PLLA have different degradation kinetics and mechanical properties. Therefore, we postulate the need for an individual approach in determining the processing window for polylactide powders. As the upper limit of the processing window, we set the beginning of melting as the temperature of the first detectable deviation from the interpolated baseline of the melting peak. Such an upper limit allows the exclusion of any thermal and structural changes of polylactide from the process window and the designated temperature range ensures a stable sintering window. The estimated sintering window ranges according to the proposed rules were experimentally verified and will be presented in our next publication.

## Figures and Tables

**Table 1:** First avalanche angle (AA), flowing angle (α_f_), cohesive index (σ_f_), and particle size distribution of the prepared PLA and PLLA powders.

Sample	AA (°)	α_f_ (°)	σ_f_ (a.u.)	d_50_ (µm)	(d_90_ − d_10_)/d_50_ (a.u.)	<10 µm (%)
		at 30 rpm (↑)				
PLA_40	33.9	55.9	24.8	36.45	1.37	3.78
PLLA_40	30.0	51.0	28.0	36.01	1.37	3.03

**Table 2:** Mass loss at 200 °C and the temperature corresponding to a 5 wt % mass loss of PLA and PLLA microspheres.

Sample	Mass Loss at 30–200 °C (wt %)	T_−5%_ (^o^C)
PLA_40	0.49	332
PLA_70	0.06	335
PLA_90	0.05	337
PLA_120	0.02	338
PLLA_40	0.25	329
PLLA_70	0.24	329
PLLA_90	0.04	329
PLLA_120	0.02	333

**Table 3:** Thermal parameters of PLA and PLLA microspheres annealed at 40, 70, 90, and 120 °C.

Sample	T_g_ (°C)	T_cc_^onset^ (°C)	T_cc_ (°C)	ΔH_cc_ (J/g)	T_m_^beginning^ (°C)	T_m_^onset^ (°C)	T_m_ (°C)	ΔH_m_ (J/g)	X_c_^DSC^ (%)
PLA_40	63	104	115	16.9	133	144	152	41.7	23.2
PLA_70	61	106	110	10.5	131	143	152	42.3	29.7
PLA_90	64	-	-	-	118	146	152	44.4	41.1
PLA_120	63	-	-	-	117	144	150	44.8	41.9
PLLA_40	61	88	94	0.7	140	174	178	65.5	45.3
PLLA_70	68	85	92	1.8	143	174	178	63.9	43.5
PLLA_90	64	-	-	-	144	174	178	53.6	37.5
PLLA_120	73	-	-	-	146	175	179	54.4	38.0

**Table 4:** Thermal resistivity and conductivity of microspheres.

Sample	S (W/K)	R (K/W)	κ (W/mK)
PLA_40	4.54 × 10^−4^	2.20 × 103	0.073
PLLA_40	4.00 × 10^−4^	2.50 × 103	0.064

## Data Availability

Data is contained within the article or supplementary material.

## Supplementary Materials

The following are available online at https://www.mdpi.com/article/10.3390/ma14112999/s1, Figure S1: Microscopic images of the powder particles of polylactide with 4 wt% of D-lactide content (PLA) (A) and poly(L-lactide) (PLLA) (B). Annealing temperatures from the left to the right: 40 °C, 70 °C, 90 °C, 120 °C, Figure S2: The TGA curves of PLA (A) and PLLA (B) microspheres annealed at 40°, 70°, 90° and 120°C, Figure S3: The cooling curves of PLA (A) and PLLA (B) microspheres annealed at 40°, 70°, 90° and 120°C, Figure S4: The first heating DSC curve of PLA_90 combined with the 1st and 2nd derivatives of the heat flow, Figure S5: The melting endotherms of indium beads placed on neat PLA and PLLA. The neat PLA and PLLA was performed by melting in DSC pan, Figure S6: Deconvolution of diffraction curves of tested samples.
