# Supplementary material for: Influence of Thermal Annealing on the Sinterability of Different Grades of Polylactide Microspheres Dedicated for Laser Sintering
Source: Materials (Basel). 2021 Jun 1;14(11):2999. doi: 10.3390/ma14112999 (PMC8198009; doi:10.3390/ma14112999)
Supplement: Supplementary file 1 [file materials-14-02999-s001.zip › materials-1168848-supplementary.pdf]

Supplementary materials

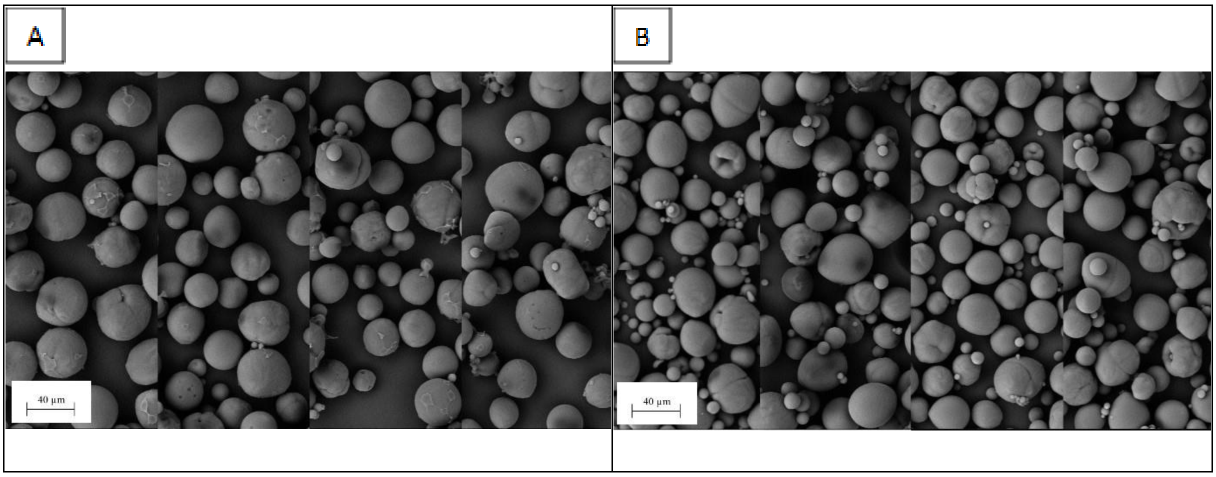

Figure S1. Microscopic images of the powder particles of polylactide with 4 wt% of D-lactide content (PLA) (A) and poly(L-lactide) (PLLA) (B). Annealing temperatures from the left to the right: 40°C, 70°C, 90°C, 120°C.

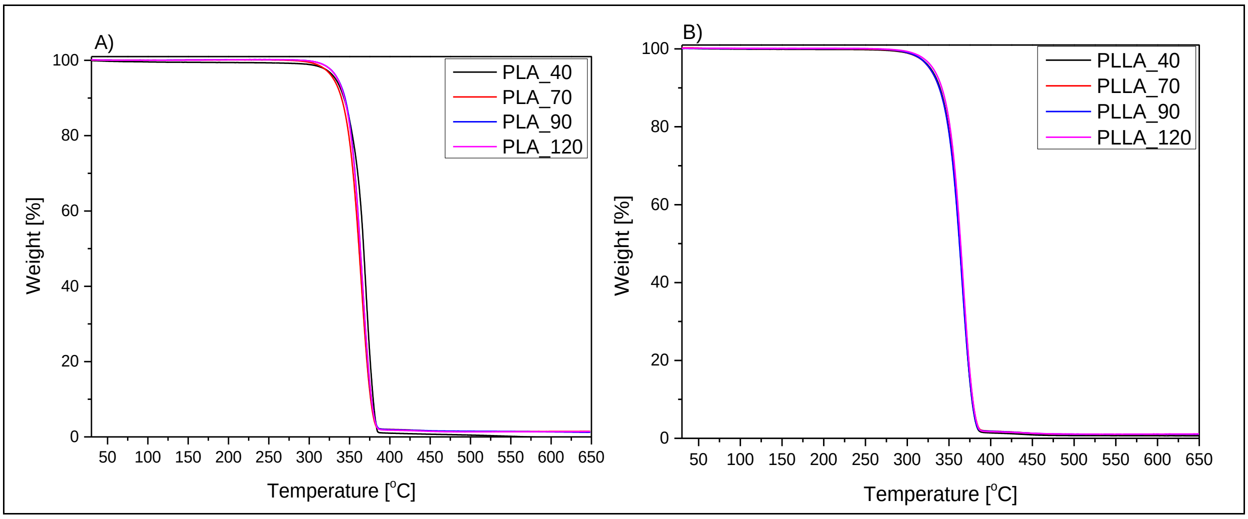

Figure S2. The TGA curves of PLA (A) and PLLA (B) microspheres annealed at 40°, 70°, 90° and 120°C.

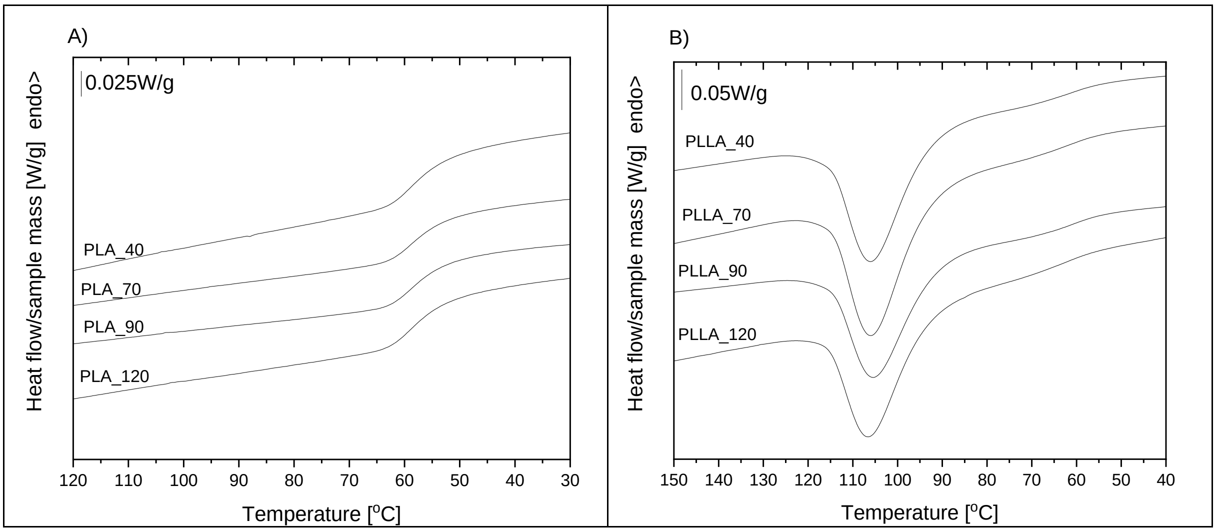

Figure S3. The cooling curves of PLA (A) and PLLA (B) microspheres annealed at 40°, 70°, 90° and 120°C.

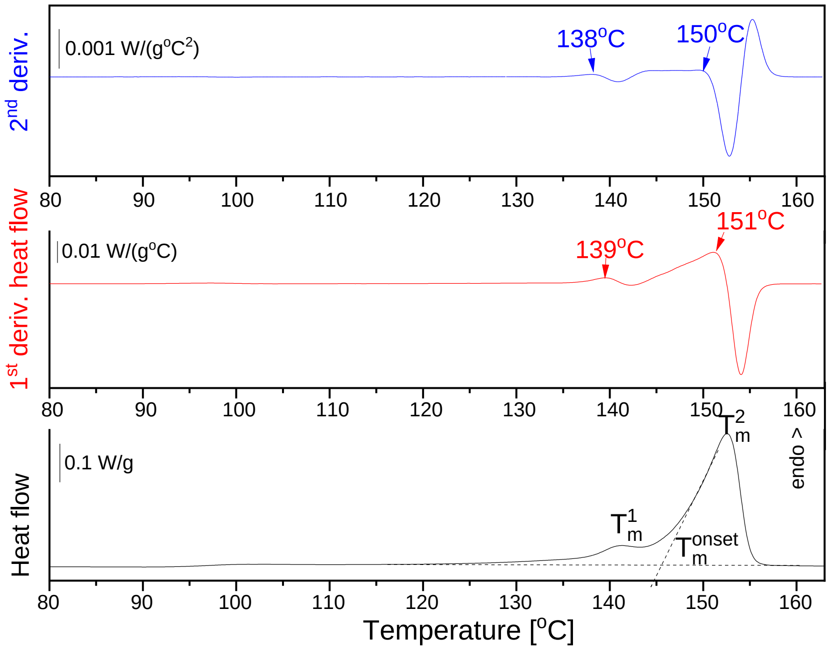

Figure S4. The first heating DSC curve of PLA_90 combined with the 1st and 2nd derivatives of the heat flow.

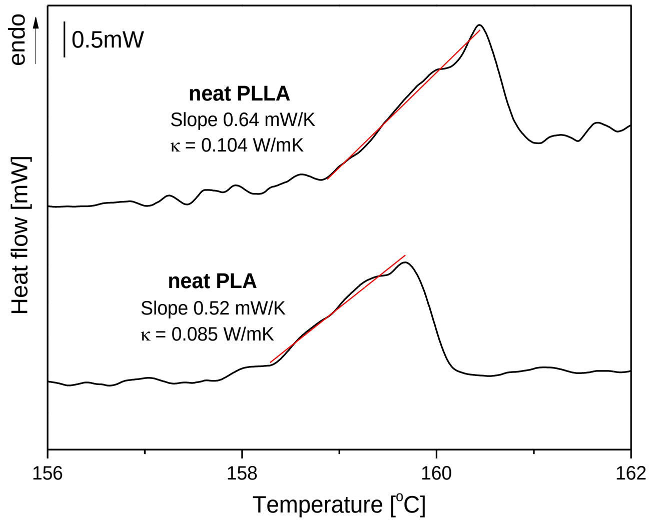

Figure S5. The melting endotherms of indium beads placed on neat PLA and PLLA. The neat PLA and PLLA was performed by melting in DSC pan.

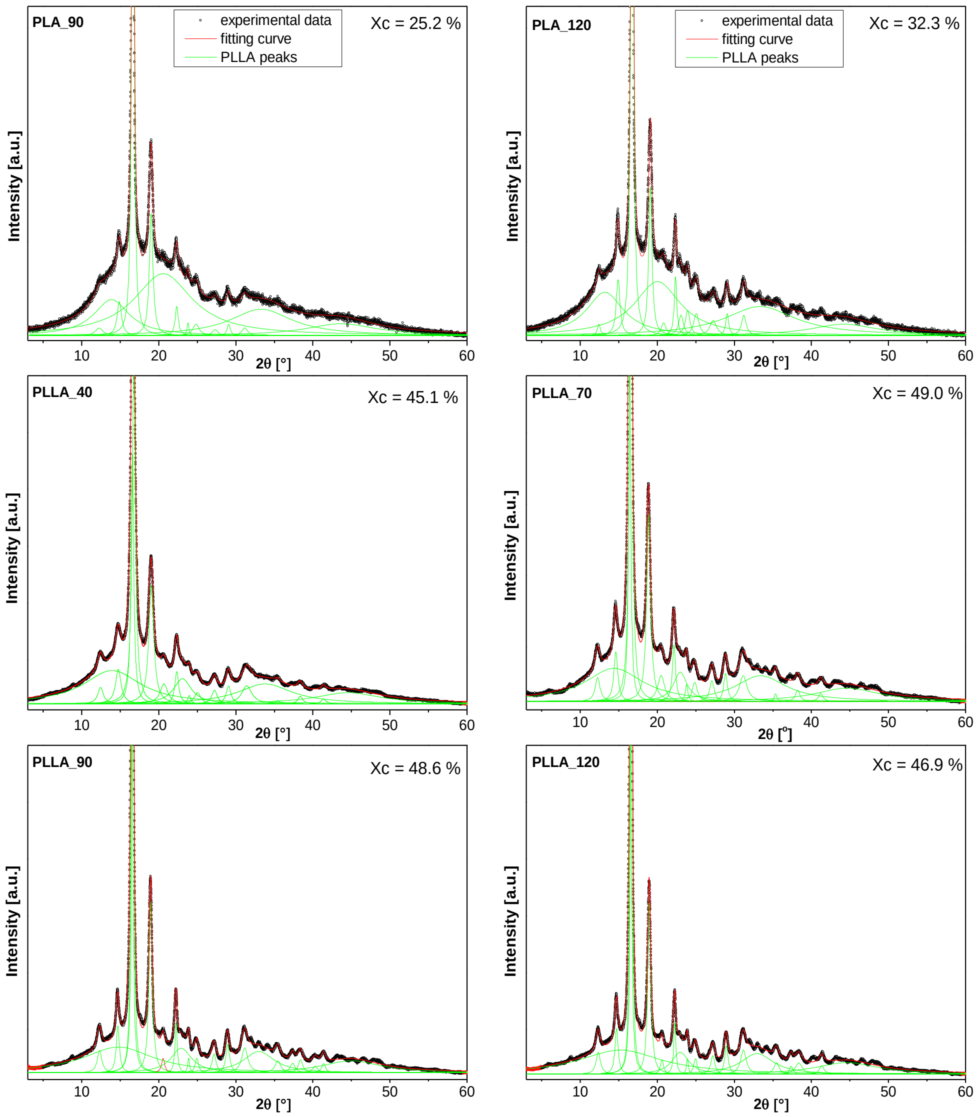

Figure S6. Deconvolution of diffraction curves of tested samples.
